# Adverse birth outcome among women who gave birth at the University of Gondar comprehensive specialized hospital, Northwest Ethiopia

**DOI:** 10.1186/s12884-024-06478-z

**Published:** 2024-04-17

**Authors:** Melak Jejaw, Getachew Teshale, Lake Yazachew, Endalkachew Dellie, Ayal Debie

**Affiliations:** 1 Department of Health Systems and Policy, Institute of Public Health, University of Gondar, P.O. BOX: 196, Gondar, Ethiopia; 2 College of Medicine and Public Health, Flinders University, Adelaide, Australia

**Keywords:** Adverse birth outcome, Low birth weight, Still birth, Preterm birth, University of Gondar comprehensive specialized hospital

## Abstract

**Background:**

In Ethiopia, various maternal and child health interventions, including comprehensive and basic obstetric cares were conducted to curb high neonatal and infant morbidity and mortality. As such, adverse birth outcome has been a public health concern in the country. Thus, this study aimed to assess the burden and associated factors with adverse birth outcomes among women who gave birth at the University of Gondar Comprehensive Specialized Hospital, Northwest Ethiopia.

**Methods:**

A health facility-based cross-sectional study was employed from 30 March to 01 May 2021 at the University of Gondar Comprehensive Specialized Hospital. A total of 455 women were interviewed using a structured questionnaire. A binary logistic regression model was fitted Adjusted Odds Ratio (AOR) with 95%CI and p-value < 0.05 were used to declare factors significantly associated with adverse birth outcomes.

**Results:**

In this study, 28% of women had adverse birth outcomes (8.4% stillbirths, 22.9% preterm births, and 10.11% low birth weights). Women aged 20–34) (AOR: 0.32, 95%CI: 0.14, 0.76), rural dwellers (AOR: 2.7, 95%CI: 1.06, 6.32), lack of ANC visits (AOR: 4.10, 95%CI: 1.55, 10.85), APH (AOR: 3.0, 95%CI: 1.27, 7.10) and fever (AOR: 7.80, 95%CI: 3.57, 17.02) were associated to stillbirths. Multiple pregnancy (AOR:7.30, 95%CI:1.75, 20.47), rural dwellers (AOR:4.60, 95%CI:1.36, 15.52), preterm births (AOR: 8.60, 95% CI: 3.88, 19.23), previous perinatal death (AOR:2.90, 95%CI:1.35, 6.24), fever (AOR:2.7,95%CI:1.17 ,6.23) and premature rupture of membrane (AOR:2.60, 95% CI:1.02, 6.57) were affecting low birth weights. In addition, previous antepartum hemorrhage (AOR: 2.40, 95%CI: 1.37, 4.10) and fever (AOR: 3.8, 95%CI: 2.13, 6.89) were also factors contributing to preterm births.

**Conclusion:**

Adverse birth outcomes continue to pose a significant public health concern. Such high rates of adverse birth outcomes, such as preterm birth, low birth weight, and birth defects, can have serious and long-lasting effects on the health and well-being of both infants and their families, and the community at large. As such, public health efforts are crucial in addressing and mitigating the risk factors associated with adverse birth outcomes. This may involve implementing interventions and policies to improve maternal health, access to prenatal care and nutritional support, and reducing exposure to environmental risks.

## Background

Pregnancy is a state of having implanted products of conception located either in the uterus or elsewhere in the body. It ends through either spontaneous or elective abortion or delivery. During this time, the mother’s body goes through immense changes involving all organ systems to sustain the growing fetus [1]. Along with this, a woman has either successful or adverse birth outcomes [2]. Adverse birth outcome is the loss of newborns during early and late pregnancy, give birth earlier than the anticipated date, and deliver low birth weight [3]. It also leads to neonatal and infant morbidity and mortality [4]. Approximately 15 million babies were born prematurely every year globally and nearly 90% and 80% of preterm occurred in Low and Middle-Income countries and sub-Saharan Africa, respectively [5]. Approximately 20 million low birth weight babies were born annually, developing countries and sub-Saharan Africa accounts 17% and 9.76% respectively. Globally, around two million babies are stillborn annually and, about 84% and 75% of stillbirth contributed by low and lower-middle income and Sub-Saharan Africa respectively. About more than 40% of stillbirth occurred immediately after onset of labor and about extra 20 million stillbirths is predicted to be occur before 2030. Preterm delivery leads to newborn under-five deaths and the survivors remain suffering from lifetime physical, neurological or educational disability with pronounced cost to families and societies. Likewise, the common cause of LBW in developed and developing countries is preterm and Intrauterine Growth Restriction (IUGR) respectively. Moreover, stillbirth leads to psychological cost and financial consequence on women, families and societies [6]. In Ethiopia and Gondar about 30% and 11.2% of the babies born with LBW respectively with rate of one infant die per ten second due LBW [7]. Birth weight is a good health indicator and determinant of the future health status of the infant’s physical, survival and mental growth. However, low attentions was given to improve birth weight in Ethiopia [8]. Besides, the magnitude of stillbirth in 2019 in Ethiopia and Gondar was 90,323 and 7.1% respectively [9].

In Prior study, extreme parity, previous history of preterm or abortion, younger maternal age, inadequate of prenatal care, antepartum hemorrhage, premature rupture of membrane and induced labor were predictors of preterm birth [9, 10]. Besides, previous study showed that prematurity, previous history of adverse birth outcome, maternal age, and lack of antenatal care (ANC) follow up, twin pregnancy, anemia and inadequate food intake during pregnancy, mothers with a history of abortion and rural residents were predictors of LBW [11, 12].

Moreover, preterm birth, advanced maternal age, history of stillbirth, extremes of neonatal birth weight, cesarean delivery, lack of antenatal care visit, assisted breach delivery and operational vaginal delivery were predictors of stillbirth [9, 13] Adverse birth outcomes are critical public health issue in Gondar (23%) [9] Generally, the global targets to reduce still birth of late third trimester is reaching 12 to fewer per 1,000 total live births in each country by 2030 [6], by develop strategic plan for maternal and child health and generate base line data for further research. Therefore, this study aimed to assess the magnitude and associated factor of adverse birth outcomes among women who gave birth at university of Gondar comprehensive specialized hospital.

## Methods

### Study design and setting

The study employed a facility based cross sectional design to assess the magnitude and factors associated with adverse birth outcome from March 30/2021 and May 1/2021 at the University of Gondar Comprehensive Specialized Hospital. University of Gondar Comprehensive Specialized Hospital is located in Gondar town, Northwest Ethiopia which is 780 kilo meters away from Addis Ababa, Ethiopia. University of Gondar Hospital was established in 1954 as a public health college and training center and currently it is the only referral teaching hospital in the Northwest Amhara and serving approximately for more than seven million people It had six departments; internal medicine, surgery, pediatrics, and Gynecology and Obstetrics. In Obstetrics department, there are a total of 42 beds (9 labor beds and 34 maternity beds), two Gynecological operational theaters, three maternity wards, one labor ward with six delivery coach and one emergency ward. According to maternity ward coordinators and, the health information system and Planners report, mostly, an average of 13 to 16 mothers per day with an estimated range of 400 to 480 women per month give birth in this comprehensive specialized referral hospital.

### Sample size and sample procedure

The total sample size was determined by using single population proportion formula by considering the proportion of adverse birth outcome of 22.7% in prior study at Gondar [14] at 95% confidence interval (CI), 4% margin of error (d) [13] and 10% of non-response rate and the final sample size was 465. Mothers who gave birth during our study period were selected consecutively until the desired sample size was obtained.

### Study population

All mothers who came for delivery service at UoGCSH were the source population and all women who gave birth at UoGCSH during study period were the study population. Critically ill women who were unable to communicate were **e**xcluded from the study. By assuming of client flow at UoGCSH is random by itself and data.

### Operational definitions

Adverse birth outcome is the presence of at least one or all of following abnormal condition [15]. Preterm birth; a baby born after 28 and before 37 completed gestational weeks [9], Low Birth Weight; the weight of the newborn less than 2500 g (5.5 pounds) regardless of gestational age, still birth; when the baby born with no sign of life (i.e. death before the complete expulsion or extraction of a product of conception from its mother) in the third trimester (≥ 28 completed weeks of gestation) or with birth weight ≥ 1000 g or length ≥ 35 cm [16] and congenital anomaly: a newborn who had any abnormality of physical structure; deviation from the normal warrant design in terms of the usual number, size, shape, location of any part, organ and cells that occurred during birth [17]. Whereas, perinatal death defined as pregnancy loss after 28 weeks of gestational age and death of newborn within the first seven days of live birth. Abortion is the termination of pregnancy or expulsion of conception from the mother in 28 weeks or fewer and weighing less than 500 gram [18].

### Data collection tools and procedures

Structured pretested interviewer administered questionnaires were adapted from different literature and modified based on the context of study area [9, 13, 19–21]. The questionnaire includes; socio demography characteristics, obstetric related factors, medical history of clients and birth outcome assessment and it was translated from English to Amharic language and back to English for the purpose of consistency. Data were collected by trained two BSc female midwives who have bachelor degree in midwifery, one BSc female nurse who have bachelor degree in nursing and supervised by a health officer for daily checkup for ensuring the completeness and consistency. One day training was provided for data collectors and supervisor in same day about the data collecting process, informed consent, technique of interview, data collection time and organization of questionnaire. Oral informed consent was taken from all study participant based on Ethiopian context of age of 18 and above years is eligible for oral informed consent and all eligible mothers who came to the hospital for delivery during data collection period were interviewed. Besides interview, the mother’s and newborn’s medical card were reviewed by the data collectors. Moreover, the trained data collectors measured and verified the newborn’s weight. The overall data collection process was supervised by the principal investigators.

### Data management and analysis

All collected questioners were rechecked for completeness and coded, and correction was made accordingly. Then the data were entered into EPI data version 4.4.1 and exported to SPSS version 20 for analysis. Descriptive statistics of frequency and cross tabulation were performed and the result was portrayed via table. Both bi-variable and multi-variable logistic regression analyses were performed to identify associated factor to adverse birth outcome. In bi-variable logistic regression analysis, variables with p-value less than 0.2 [13] were included in multivariable logistic regression, were fitted for the three major adverse birth outcomes separately, to control the possible effect of confounder. Adjusted odd ratio (AOR) with 95% confidence interval (CI) and p-value < 0.05 were used to declare statistically significant variable with adverse birth outcome. The hosmer-lemeshow goodness-of-fit statistic was carried out to assess the fulfillment of the basic assumption of multiple logistic regressions and the model was good which had p-value > 0.05.Those newborns that had low birth weight, congenital anomaly and preterm delivery were linked to neonatology ward for further evaluation and additional neonatal service. Mothers who had adverse birth outcome (still birth for the current pregnancy) were reassured and counseled to have strict ANC follow up for those who have plan to get pregnancy in the future.

## Results

### Study characteristics

A total of 455 mothers were interviewed with response rate of 97.8%. The mean age of the women was 30.3(SD ± 4.9) years. More than half, 56.7%( 258) of mothers were urban dwellers and majority, 80.9%( 368) of mothers were Orthodox Christian followers. Nearly, one third 33.4% (152) of the mothers were not attending formal education and about 47% (214) of mothers were house wife (Table 1).

**Table 1:** Socio-demographic characteristics of mothers who gave birth at UoGCSH, Northwest Ethiopia, 2021 (*n* = 455)

Variables	Category	Frequency(*n* = 455)	Percent (%)
Age	18–20	27	6.0%
	20–34	331	72.7%
	≥ 35	97	21.3%
Residence	Urban	258	56.7%
	Rural	197	43.3%
Religious	Orthodox	368	80.9%
	Muslim	67	14.7%
	Protestant	20	4.4%
Maternal educations status	No formal education	152	33.4%
	Primary level	79	17.4%
	Secondary level	108	23.7%
	College and above level	116	25.5%
Maternal occupational status	House wife	214	47%
	Merchant	77	17%
	Governmental employer	120	26.4%
	NGO	27	5.9%
	Others	17	3.7%
Marital status	Single	57	12.53%
	Married	335	73.63%
	Divorced	63	13.84%
Ethnicity	Amhara	355	78%
	Qimant	100	22%
Age at 1st marriage	< 18	17	3.7%
	Above 18	438	96.3%
Family size	≤ 5	406	89.2%
	Above 5	49	10.8%

Others* refers to daily laborers and daily help

### Obstetric characteristics of study participants

Majority, 90.8% (413) of the mothers were attended ANC follow up for the current pregnancy. About 71.3%( 295) of women started their ANC follow up during the first trimester of pregnancy and about 36.8% (152) of them had at least four ANC visit during their recent pregnancy period. In addition, more than two-third 68.8% (313) of mother got dietary counseling during ANC visit (Table 2).

**Table 2:** Obstetrics related characteristics of mothers who gave birth at UoGCSH, Northwest Ethiopia, 2021 (*n* = 455)

Variable	Category	Frequency	Percent
ANC follow up status	Yes	413	90.8%
	No	42	9.2%
Number of ANC visits(*n* = 413)	1 times	25	6.1%
	2–3 times	236	57.1%
	≥ 4 times	152	36.8%
Time of 1st ANC Visit(*n* = 413)	First trimester	295	71.4%
	Second trimester	92	22.3%
	Third trimester	26	6.3%
Maternal gravidity	Prim gravida	87	19.1%
	Multigravida	368	80.9%
Modern contraceptive use prior to current Pregnancy	Yes	299	65.7%
	No	156	34.3%
Types of contraceptive used(*n* = 299)	Pills	148	49.5%
	Injectable	98	32.8%
	Implanon	36	12%
	IUCD	17	5.7%
Dietary counseling during pregnancy	Yes	313	68.8%
	No	142	31.2%
Mode of delivery	SVD	292	64.2%
	CS	128	28.1%
	Instrumental delivery	35	7.7%
sex of the current baby	Male	133	29.2%
	Female	322	70.8%
Labor duration	≤ 9.4 h	323	71%
	> 9.4 h	132	29%
Labor status	Spontaneous	357	78.5%
	Induced	98	21.5%
Birth space in years	< 3	108	23.7%
	3–4	262	57.6%
	5+	85	18.7%
history of perinatal death	Yes	103	22.6%
	No	352	77.4%
Congenital malformation	Yes	18	4%
	No	437	96%
PROM in this pregnancy	Yes	43	9.5%
	No	412	90.5%
APH in this pregnancy	Yes	84	18.5%
	No	371	81.5%
Pregnancy status	Planed and wanted	298	65.5%
	Unplanned but wanted	126	27.7%
	Unplanned and unwanted	31	6.8%
Gestational age of the current pregnancy	Preterm pregnancy	132	29%
	Term pregnancy	323	71%

### Medical and other obstetrics related characteristics

About 22%( 100) mothers had medical illness and 59%( 59) of them had Anemia. In addition, one from six (16.9%) and 18.7% mothers, had history of fever and hypertension respectively during the recent pregnancy. Besides, majority, 96.5%( 439) of the participants was screened for HIV and only 3.2% of them were found to be HIV seropositive. However; only 78.6% (11) of HIV confirmed participants start Anti-retroviral treatment (Table 3).

**Table 3:** Medical and other obstetrics related characteristics of mothers who gave birth at UoGCSH, Northwest Ethiopia, 2021 (*n* = 455)

Variable	Category	Frequency	Percent
Adverse birth outcome (at least one	Yes	127	27.9%
	No	328	72.1%
All 3 key adverse birth outcomes	Yes	7	1.5%
	No	448	98.5%
Birth Weight	Low birth weight	46	10.11%
	Normal	409	89.9%
Birth outcome of this pregnancy	Sill birth	38	8.4%
	Live birth	417	91.6%
Medical illness	Yes	100	22%
	No	355	78%
Types of medical illness(*n* = 100)	Anemia	59	59%
	Covid19	15	15%
	UTI	14	14%
	Malaria	12	12%
Fever (≥ 2 weeks)	Yes	77	16.9%
	No	378	83.1%
Hypertension	Yes	85	18.7%
	No	370	81.3%
HIV screening status	Yes	439	96.5%
	No	16	3.5%
HIV test result(*n* = 439)	Positive	14	3.2%
	Negative	425	96.8%
ART status(*n* = 14)	Started	11	78.6%
	None	3	21.4%
Physical harassment	Yes	12	2.6%
	No	443	97.4%
Time to take to reach the hospital	≤ 30 min	181	39.8%
	> 30 min	274	60.2%

### Prevalence and associated factors of still birth

The prevalence of still birth was 8.4% (95% CI: 6%, 11%). The odd of women whose age 20 to 34 years old were 68% lower to have adverse birth outcomes of still birth as compared to women whose age above 35 years (AOR = 0.32;95%CI: 0.14, 0.76). In addition, rural dwellers women were 2.7 times higher to have an adverse birth outcomes of still birth as compared to counter parts (AOR = 2.7; 95%CI:1.06, 6.32). On the other hand, mothers who had no antenatal care follow up were 4.1 times more likely to develop still birth as compared with mother who had antenatal care follow up during pregnancy period (AOR = 4.1; 95%CI:1.55, 10.85). Besides, mothers who gave prematurity birth were 2.1 times high risk to have adverse birth outcome of still birth as compared to term birth (AOR = 2.1; 95%CI:1.09, 4.6). Moreover, the odds of women who had antepartum hemorrhage during pregnancy were 3 times higher to have still birth as compared to counterparts (AOR = 3.0; 95%CI: 1.27, 17.02). Furthermore, mothers who had fever of ≥ 2 weeks during pregnancy were 7.8 times more likely to have adverse birth outcome of still birth as compared to counterparts(AOR = 7.8; 95%CI: 3.57, 17.02) (Table 4).

**Table 4:** Factor associated with still birth among deliveries in UoGCSH, Northwest Ethiopia (*n* = 455), 2021

Variable	Still Birth		Crude OR (95% CI)	Adjusted OR (95% CI)
	Yes	No		
**Age**				
≤ 20	2	25	0.52(0.11–2.45)	0.24(0.04–1.51)
20–34	23	308	0.48(0.23–0.99)	0.32(0.14–0.76)*
≥ 35	13	84	1	1
**Mothers educational level**				
No formal education	20	132	3.4(1.22–9.25)	1.13(0.2–6.34)
Primary level	9	70	2.9(0.92–8.87)	1.6(0.35–7.17)
Secondary level	4	104	0.85(0.22–3.27)	0.69(0.16–3.06)
College and above	5	111	1	1
**Residence**				
Rural	27	170	3.6(1.72–7.38)	2.7(1.06–6.32)*
Urban	11	247	1	1
**Sex**				
Male	18	115	2.4(1.21–4.62)	1.9(0.85–4.14)
Female	20	302	1	1
**ANC follow up status**				
No	13	29	6.9(3.22–15.01)	4.1(1.55–10.85)*
Yes	25	388	1	1
**Gestational age**				
Preterm pregnancy	132		3.4(1.73–6.69)	2.1(1.09–4.60)*
Term pregnancy	323		1	1
**Birth weight**				
Low birth weight	4	23	2.7(0.97–7.65)	1.5(0.47–4.95)
Normal birth weight	29	399	1	1
**History of perinatal death**				
Yes	9	94	1.65(0.80–3.41)	1.1(0.45–2.58)
No	24	328	1	1
**APH**				
Yes	11	73	1.92(0.91–4.04))	3.0(1.27–7.10)*
No	27	344	1	1
**PROM**				
Yes	5	38	2.39(0.98–5.81)	1.3(0.44–3.81)
No	28	384	1	1
**Fever (≥ 2 weeks)**				
Yes	21	56	7.96(3.96–16.02)	7.8(3.57–17.02)*
No	17	361	1	1

*Significantly associated factors at a p-value < 0.05

### Prevalence and factors associated with low birth weight (LBW)

The prevalence of low birth weight was 10.11% (95% CI: 7%, 13%). The mean weight of the newborn was 3,096.7 (SD ± 436.21) grams.

From fitted multivariable logistic regression model factors such as rural resident, multiple pregnancy, perinatal history, had fever and PROM were associated with low birth weights.

The odds of low birth weight among mothers from rural areas were 4.6 times higher compared to urban resident (AOR = 4.6, 95% CI: 1.36, 15.52). Similarly, the odds of adverse birth outcomes of low birth weight among preterm birth was 8.6 times higher compared to counterparts of term pregnancy (AOR = 8.6, 95% CI: 3.88, 19.23). Likewise, the odds of low birth weight among mothers who had multiple pregnancies were 7.3 times higher compared to women who had singleton (AOR = 7.3, 95% CI: 1.75, 20.47). Moreover, the odds low birth weight among women who had history of perinatal death were 2.9 times higher to have adverse outcome of low birth weight when compared to those without history of perinatal death (AOR = 2.9, 95%CI: 1.35, 6.24). Furthermore, women who had fever of more than 2 weeks were 2.7 times more likely to have adverse birth outcomes of low birth weight as compared to the counterparts of without fever of more than 2 weeks (AOR = 2.7, 95% CI: 1.17, 6.23) and the odds of adverse birth outcomes of low birth weight was higher among women who developed premature rupture of membrane compared to counterparts (AOR = 2.6, 95% CI: 1.02, 6.57) (Table 5).

**Table 5:** Bi-variable and multi-variable analyses of factors associated with low birth weight among deliveries in UoGCSH, Northwest Ethiopia (*n* = 455), 2021

Characteristics	Low birth weight		Crude OR (95% CI)	AOR (95% CI)
	Yes	No		
**Residence**				
Urban	18	240	1	1
Rural	28	169	2.21(1.18–4.12)	4.6(1.36–15.52)*
**Mothers educational level**				
No formal education	20	132	1.8(0.79–4.12)	0.25(0.05–1.18)
Primary level	10	69	1.72(0.67–4.46)	0.97(0.27–3.47)
Secondary level	7	101	0.82(0.29–2.29)	0.78(0.24–2.51)
College and above	9	107	1	1
**Family size**				
≤ 5	45	361	1	1
> 5	1	48	0.17(0.02–1.24)	0.07(0.01–1.64)
**Pregnancy type**				
Singleton	41	399	1	1
Multiple	5	10	4.9(1.59–14.9)	7.3(1.75–20.47)*
**Gestational age**				
Preterm pregnancy	34	98	8.9(4.48–18.03)	8.6(3.88–19.23)*
Term pregnancy	12	311	1	1
**History of perinatal death**				
Yes	18	85	2.5(1.29–4.64)	2.9(1.35–6.24)*
No	28	324	1	1
**Fever (≥ 2 weeks)**				
Yes	14	63	2.4(1.21–4.76)	2.7(1.17–6.23)*
No	32	346	1	1
**Medical illness**				
Yes	14	86	1.64(0.84–3.22)	0.9(0.38–1.99)
No	32	323	1	1
**Congenital anomaly**				
Yes	4	14	2.69(0.85–8.54)	3.2(0.71–14.38)
No	42	395	1	1
**APH**				
Yes	14	70	2.11(1.07–4.17)	2.0(0.9–4.51)
No	32	339	1	1
**PROM**				
Yes	10	33	3.17(1.44–6.94)	2.6(1.02–6.57)*
No	36	376	1	1

### Prevalence and factors associated with preterm birth

The prevalence of preterm delivery was 22.9% (95% CI: 19%, 27%). The mean gestational age was 37.3(SD ± 1.6) weeks. From multivariable logistic regression model variables whose p value < 0.05 were fever and antepartum hemorrhage. However; as shown in the logistic regression analysis, pregnancy type, lack of antenatal care follow up, birth space, medical illness, perinatal death history and premature rupture of membrane were associated with preterm birth in binary logistic analysis and turned out in multi-variable analysis.

The odds of preterm birth of mothers who had fever of two and more weeks were 3.8 times higher had adverse birth outcomes of preterm delivery as compared to counter parts (AOR 3.8, 95% CI; 2.13, 6.89). Similarly, women who developed antepartum hemorrhage were 2.4 times more likely to have adverse birth outcomes of preterm birth as compared to those women who did not developed antepartum hemorrhage (AOR: 2.4, 95% CI; 1.37, 4.10) were significantly and independently associated with preterm birth (Table 6).

**Table 6:** Bi-variable and multivariable analyses of factor associated with preterm birth among deliveries in Gondar University Comprehensive specialized Hospital, Northwest Ethiopia (*n* = 455), 2021

Characteristics	Preterm birth		Crude OR (95% CI)	Adjusted OR (95% CI)
	Yes	No		
Pregnancy type				
Single	125	315	1	1
Multiple	7	8	2.2(0.78–6.21)	2.6(0.86–7.69)
**Number of ANC visits**				
1 times	4	21	2.4(0.21–1.97)	2.1(0.67–6.58)
2–3 times	73	163	1.6(0.94–2.39)	1.4(0.43–4.54)
≥ 4 times	35	117	1	1
**History of perinatal death**			1	
Yes	36	67	1.4(0.9–2.29)	0.9(0.49–1.58)
No	96	256	1	1
**Birth space**				
< 3	27	81	1	1
3–4	84	178	1.0(0.53–1.96)	1.0(0.48–2.15)
5+	21	64	1.4(0.82–2.51)	1.3(0.67–2.39)
**Medical illness**				
Yes	38	62	1.7(1.1–2.72)	1.2(0.72–2.17)
No	94	261	1	1
**Fever ≥ 2 weeks**				
Yes	43	34	4.1(2.5–6.8)	3.8(2.13–6.89)**
No	89	289	1	1
**Antepartum Hemorrhage**				
Yes	34	50	1.9(1.16–3.1)	2.4(1.37–4.10)**
No	98	273	1	1
**Premature rupture of membrane**				
Yes	17	26	1.7(0.88–3.23)	1.6(0.74–3.40)
No	115	297	1	1

*Significantly associated factors at a p-value < 0.05

## Discussion

The present study showed the prevalence and associated factors of adverse birth outcome among mothers who gave birth at University of Gondar Comprehensive Specialized Hospital. Maternal factors such as age, residence, antenatal care follow-up, preterm delivery, antepartum hemorrhage, having fever ≥ two weeks, multiple pregnancy, perinatal death history and premature rupture of membrane were statistically significant predictors of adverse birth outcome.

In this study prevalence of adverse birth outcome was found to be high. This finding was consistent with report of similar study done in North Wollo [18] and pooled prevalence of Sub-Saharan Africa [22]. However; this result was lowered as compared to similar study done in Wollo [13] and in Gamo Gofa [20]. The difference could be study participants variation. Most of study participants in Gamo Gofa were rural dwellers indeed, rural dwellers women are more likely to have adverse birth outcome due to women from rural dwellers are more exposed to social restrictions, unemployment, overwork, literacy, lack of health care decision making participation and access to maternal health service than women of urban dwellers [23]. This implies urban dwellers of women have low influence on adverse birth outcome. The other possible reason for the discrepancy might be in Gamo Gofa, majority (94.5%) women gave a birth within before seven month of gestation [20] whereas in this study around three-fourth of birth were preterm deliveries since preterm birth are greatly contributed to adverse birth outcome [24].

On the other hand, this findings’ of adverse birth outcome was higher as compared to study done in Hosanna, Ethiopia [25] and Suhul hospital Tigray [26]. The possible justification for the discrepancy might be study area difference. The former studies included general hospital. Majority rural women give birth at health centers which located in a rural area to provide services to rural women who are usually unemployed, overworked and have poor access to antenatal care, labor, and delivery services and when those women develop obstetric complication usually they were referred to referral hospital [26]; since this study was conducted in comprehensive specialized hospital in which referral case are more predominant, that increase adverse birth outcome cases at referral hospital than general hospital. This implies attention has to give for general hospital to decrease referral case and timely management of adverse birth outcome. The other reason for the variation might be due to study participant’s characteristics difference. In this study, only half percent of women who had adverse birth outcome didn’t have ANC follow-up visit for the recent pregnancy whereas study in Hosanna, at Negest Elene Mohammed Memorial General Hospital and Tigray Suhul hospital revealed that only about 16.2% and 18% of women who had adverse birth outcome didn’t have ANC follow-up visit respectively. Scholars suggested that lack of ANC follow-up visit leads to adverse birth outcome [25, 26]. This indicates great emphasis has to offer to health centers and general hospitals to provide focused ANC follow up to curb adverse birth outcome.

The prevalence of still birth was 84 per 1,000 total births. This finding is congruent with similar study done in Dessie referral hospital [18], Amhara Region [27], Negest Elene, General Hospital in Hosanna Town [25] and Southern Ethiopia [28]. However; this result was higher as compared study done in Amhara region [29] Ghana [30], Systematic review in Sub Saharan Africa [31], Axum [32], Niger [33] Tanzania [34] and Hiwot Fana Specialized University Hospital, Ethiopia [35]. The possible explanation for the variation might be maternal health service, health facility, logistic parameters, methodological, community awareness and socio-cultural factors. Since this study was conducted in referral hospital, whereas the worldwide annual report of still birth rate comprising communities and it is believed that most normal deliveries carried out in health center whereas women who experienced obstetric complication referred to referral hospital that contribute to a higher rate of adverse birth outcome at referral hospitals. In addition, ,delays from pregnant women’s health seeking behaviors due to lack of awareness and cultural restriction, and weak referral system in primary hospital and health centers attributed to increased rate of still birth in the referral comprehensive hospital even though better health services and high skilled professional avail in the referral comprehensive hospital [36].

On the other hand, this finding’ of still birth rate was lowered as compared to the report of Southeast Asia (48.5%) [22]. The discrepancy might be due to most still birth in the community were under-reported in this study even it is common problems in SSA including Ethiopia [37] whereas study done in Southeast Asia was community based. In general, the inspiring to have evidence about still birth is due to the fact that most still births are preventable through strict antenatal care follow-up and its intervention [38].

The current study prevalence of preterm birth was found to be 22.9% which was higher as compared to study done in Iran [38] Tanzania [39], Gondar Ethiopia [9] and Dessie Ethiopia [18]. The difference might be due to the variation of methodological, study population, study period and working set up.

Furthermore; the magnitude of low birth weight was found to be 10.11% which was consistent with previous study done in northern Tanzania [40], Dangla, Ethiopia [8], Ghana [41], the pooled prevalence in Sub-Saharan Africa [42] and United Arab Emirates [43]. However; it was lower than previous study done in Gondar [44].

This study finding revealed that women with middle aged 20–34 years have lower odds to have adverse birth outcome particularly still birth as compared to those women whose age above 35 years. This finding was in line with study conducted in Wollo [13], Hawassa [45] and Ghana [41]. Study done in Hawassa, Ethiopia showed that women whose age group 35–45 years were two fold high risk than those women in age group 20–34 years [45]. The possible explanation for this variation might be due to the fact that young women who got pregnancy for the first time are higher early seeker of antenatal and medical care than counterparts. In addition, coupled with age 20–34 years women may have good chance to have maternal nutrition, socio-economic status and sufficient ANC attendance. Moreover, non-modifiable risk factors, advanced maternal age had higher odds to have adverse birth outcome [25].

The odds of women with rural residents to give low birth weight newborn were four folds higher as compared to those women with urban residents. It was in line with study done in Gondar [9], Gamo Gofa [20], and Hosana town [46] and Wollo [13]. Report from Wollo revealed that the odds of women with rural residents to have adverse birth outcome was two times higher than urban residents [47]. The discrepancy might be due to variation in cultural taboos, education, overwork, unemployment, healthcare decision making and access to maternal health service. Most women in rural areas are commonly affected by the cultural/ traditional taboos on nutritional practice via inhibition; in contrary urban dwellers women have better lifestyle of balance diet practice. Urban resident women have more chance to access health facility visit and more informed about pregnancy, labor and delivery because they obtain maternal health education via different media than rural dwellers women [13]. Similar study done in Ghana [30] and Hosanna Ethiopia [25] revealed that women from rural dwellers increase risk of low birth weight. Likewise, study undertaken in Gamo Gofa Zone showed that rural women are exposed to home level overwork that attributed to adverse birth outcome [20]. Moreover; women in urban areas have higher participation in decision making of health care seeking than rural dwellers. Demography health survey of Sub Saharan Africa report showed that women with lack of participation in health care decision making were high risk to have adverse birth outcome than women participated in decision making due to the fact that women failed and low involvement in healthcare decision making was linked to low utilization of antenatal care and institutional delivery and this greatly contributed to adverse birth outcome [48].

Besides, women who didn’t have ANC visit for the recent pregnancy have high adverse birth outcome than those ANC service users. This result was consistent with study carried out in Wollo [13], Hawasa, Ethiopia [15], in Dessie referral Hospital, Ethiopia [18] in Dilla Town, Southern Ethiopia [12], systematic review and meta-analysis [49] and Sub Saharan Africa report showed that women who had ANC follow-up visit for current pregnancy were less likely to have adverse birth outcome than counterparts of women who had no follow-up visits [22]. The possible explanation for the difference might be due to ANC checkup helps to identify high risk pregnancies like intrauterine growth restriction, to received information related to nutritional counseling and supplementation of nutrient fortified foods. Besides, ANC follows up visits provide opportunity to identify disease like HIV/AIDS, syphilis, malaria and intestinal helminthiasis infection might greatly affect fetal birth outcome. Furthermore, hypertension during pregnancy might be responsible for preterm deliveries and immature newborn that could attribute to still birth and ANC follow up help to early identification of women with hypertension and take appropriate intervention to control it [50]. In general, regular ANC follow up visit allowed a chance for pregnant women to seek early treatment for those high potential pregnancy related health problems and provide access of preconception care intervention although, WHO recommended preconception care, it is not yet launched and implemented in Ethiopia health care system [51]. Hence, promote enhancement of quality of ANC and mobilization of pregnant women based on WHO current recommendations of focused care approach and preconception care intervention for pregnant women to decrease adverse birth outcome and ensuring the attainment of sustainable development goals [45].

In addition, gestational age was another independent factors affecting adverse birth outcome particularly; still birth and low birth weight. In the current study, the odds of preterm newborn to end-up with still birth were two folds higher as compared to term deliveries. Previous study finding in Gondar [9], Jimma Jone, Southwest Ethiopia [52], Dangla, Ethiopia [8], systematic review and meta-analysis [49], and Sub Saharan Africa [53] supported this finding. Study done in Gondar showed that preterm newborn were six times more likely to be born as stillbirth. Mostly, preterm newborn are immature and unable to survive until birth [54].

Women with current pregnancy complication (premature rupture of fetal membrane and antepartum hemorrhage) and history of fever more than two weeks were found to have higher risk of experiencing adverse birth outcomes( low birth weight, preterm delivery and still birth) as compared to those without pregnancy complication. This result was congruent with similar study conducted in China [55], Iran [56], Zambia [50] and, previous study of Gondar, Ethiopia [9],. Previous study in Gondar showed that pregnant women that encountered antepartum hemorrhage during pregnancy were eight folds high risk to had still birth as compared to women without antepartum hemorrhage [9]. The plausible explanation might be due to the fact bleeding during pregnancy causes anemia that leads to intra-uterine oxygen deprivation and this greatly affect the well-being of fetus in the uterus [57].

Moreover, women with multiple pregnancies had higher odd of low birth weight as compared to those women of singleton births. This finding agreed with study done in northwest Ethiopia [58] Jimma Zone, Southwest Ethiopia [52] and in Sub-Saharan Africa [22]. The possible explanation might be due to the fact that intrauterine growth restrictions, birth defects, and mechanical factors (obstructed labor, uterine rupture, mal-presentations, and mal-position) are more common in multiple pregnancies that increased the risk of still birth. Hence, twin pregnancy is referred as high-risk pregnancy that needs great attention, and birth and complication preparedness counseling should be given for pregnant women [22].

Furthermore, women who had history of perinatal death had three fold odds to have low birth weight than those women who never had history. This finding was supported by similar study report in Hawassa [45] and Shire town, north Ethiopia [26], portrayed that women who had history of child related abnormal birth outcome are more likely to have adverse birth outcome. In the recent study, about 22.9% (103) women had history of perinatal death. There is well-known evidence that showed women having history of previous abnormal birth outcome are more likely to have abnormal birth outcome in subsequent pregnancies. Evidence-based clinical practice revealed that preconception maternal care intervention provides the chance for health care providers to screen out such risk factors prior to high risk women gets conception and it reduced occurrence of similar abnormal birth outcome in the subsequent pregnancies [51]. .

## Conclusion

The overall prevalence of adverse birth outcome (still birth, preterm birth and low birth weight) at University of Gondar Comprehensive Specialized Hospital was high and still it needs an attention. Maternal age, residence, ANC follow-up, preterm birth, antepartum hemorrhage, fever ≥ two weeks, multiple pregnancy, perinatal death history and premature rupture of membrane were statistically significant predictors of adverse birth outcome. Therefore, strengthen focused ANC follow-up coverage and mobilizations of all pregnant women for timely focused care approach as per WHO recent recommendation to decrease adverse birth outcome. In addition, set strategies and policies to early detection and management of pregnancy related complication and identification of high risk women who had history of perinatal death, preterm birth, multiple pregnancies and having fever during pregnancy and advanced age pregnant women by ensuring of the availability and utilization of comprehensive obstetric and newborn care are important recommendation.

### Limitations of the study

Since this study was employed in the referral hospital, it may not show the real picture of these adverse birth outcomes in the study area. Besides, recall bias might be another limitation while determining the gestational age of women may be another possible limitation.

## Data Availability

Availability of data and materialData will be available upon reasonable request from the corresponding author.

## Abbreviations

ANC: antenatal care
AOR: adjusted odd ratio
ART: anti-retroviral therapy
APH: antepartum hemorrhage
CI: confidence interval
IUGR: intrauterine growth restriction
PROM: premature rupture of membrane
SDGs: sustainable development goal
SPSS: Statistical Package for Social Sciences
SSA: sub Saharan Africa
UoGCSH: university of Gondar comprehensive specialized hospital
